# Hierarchical Structure with Highly Ordered Macroporous-Mesoporous Metal-Organic Frameworks as Dual Function for CO_2_ Fixation

**DOI:** 10.1016/j.isci.2019.05.006

**Published:** 2019-05-09

**Authors:** Zhenxing Li, Xiaofei Xing, Dong Meng, Zhengxu Wang, Jingjing Xue, Rui Wang, Junmei Chu, Mingming Li, Yang Yang

**Affiliations:** 1 State Key Laboratory of Heavy Oil Processing, Institute of New Energy, China University of Petroleum (Beijing), Beijing 102249, China; 2 Department of Materials Science and Engineering, University of California, Los Angeles, CA 90095, USA; 3 California NanoSystems Institute, University of California, Los Angeles, CA 90095, USA

**Keywords:** Chemical Reactions in Materials Science, Chemical Synthesis, Materials Characterization Techniques

## Abstract

As a major greenhouse gas, the continuous increase of carbon dioxide (CO_2_) in the atmosphere has caused serious environmental problems, although CO_2_ is also an abundant, inexpensive, and nontoxic carbon source. Here, we use metal-organic framework (MOF) with highly ordered hierarchical structure as adsorbent and catalyst for chemical fixation of CO_2_ at atmospheric pressure, and the CO_2_ can be converted to the formate in excellent yields. Meanwhile, we have successfully integrated highly ordered macroporous and mesoporous structures into MOFs, and the macro-, meso-, and microporous structures have all been presented in one framework. Based on the unique hierarchical pores, high surface area (592 m^2^/g), and high CO_2_ adsorption capacity (49.51 cm^3^/g), the ordered macroporous-mesoporous MOFs possess high activity for chemical fixation of CO_2_ (yield of 77%). These results provide a promising route of chemical CO_2_ fixation through MOF materials.

## Highlights

• Macro-, meso-, and microporous structures were integrated in one framework
• Macroporous-mesoporous MOFs are prepared by co-assembly double template method
• Macroporous-mesoporous MOFs possess high activity for chemical fixation of CO_2_

## Graphical Abstract

**Figure:**
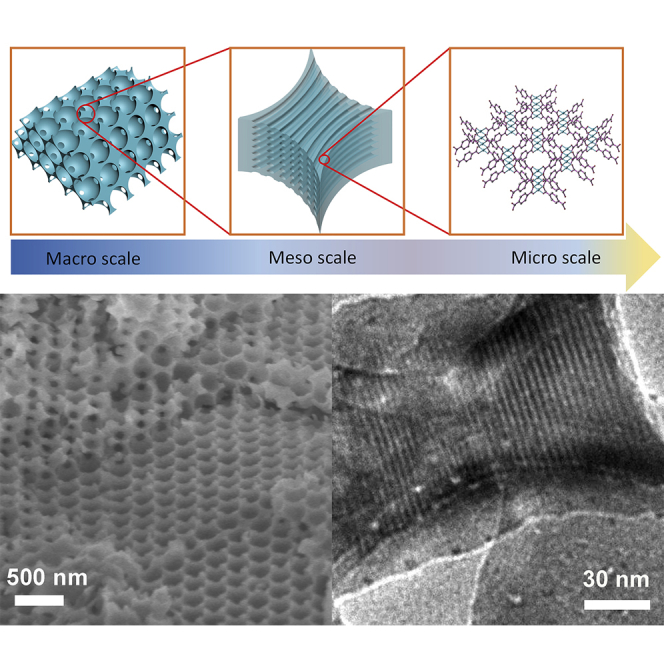

## Introduction

From natural zeolite to meso- and macroporous materials, porous materials have been extensively applied in ion exchange (Okada et al., 2015, Li et al., 2018, Qi et al., 2015), separation (Gupta et al., 2017, Chu and Pan, 2012, Dumee et al., 2013), catalysis (An et al., 2014, Sun et al., 2016, Qian et al., 2009), drug delivery (Wang et al., 2017, Shin et al., 2017), and other fields (Kitagawa et al., 2004). The skeleton of early porous materials was composed of inorganic compounds. A new type of nanoporous material, metal-organic frameworks (MOFs), which has the properties of both inorganic and organic materials, has drawn great attention in recent years (Yaghi et al., 1995, Yaghi et al., 2003, Yaghi and Li, 1995, Matsuda et al., 2005, Lin et al., 2016). MOF is a coordination polymer composed of inorganic metal structural units and organic ligands through covalent or ionic covalent bonds. Owing to their unique high specific surface area and adjustable pore structure (Furukawa et al., 2010, Férey et al., 2005, Aijaz et al., 2014), as well as diverse structure and excellent catalysis performance, MOFs have been widely used as functional materials in the areas of selective catalysis (Liu et al., 2014, Kornienko et al., 2015, Huang et al., 2018, Czaja et al., 2009), gas storage (Ma and Zhou, 2010, Yoon et al., 2013), optoelectronic materials (Chen et al., 2007, Allendorf et al., 2009, Stavila et al., 2014, Choi et al., 2014, Kong et al., 2016, Avci et al., 2018, Guan et al., 2017, Zheng et al., 2017), drug controlled release (Horcajada et al., 2010, Taylor-Pashow et al., 2009, Zheng et al., 2016), and molecular separation (Kang et al., 2014, Zhang et al., 2016; Zheng et al., 2017, Liu et al., 2018). Despite these excellent properties, most of the currently reported MOFs only have microporous regime, which severely obstructs the mass transfer and limits the access of large molecules to the active site (Furukawa et al., 2014, Xuan et al., 2012). Therefore it is significant to develop an efficient way to introduce larger pores into MOFs. Shen et al. (Shen et al., 2018) prepared ordered macroporous-microporous MOF single-crystal materials with the heterogeneous solvent-induced heterogeneous nucleation method using highly ordered polystyrene (PS) microspheres as template. Koo et al. (Koo et al., 2017) synthesized water-stable hierarchical porous MOFs by a selective acid etching process. Ma et al. (Ma et al., 2012) presented mesoporous MOFs by a rational bottom-up method using complicated disulfonate acids as ligands and metal nitrates or chlorides as inorganic precursors at high pressure and high temperature. Zhao et al. (Zhao et al., 2011) proposed the synthesis of MOF nanosphere with well-ordered mesopores in ionic liquid, high pressure, and supercritical CO_2_ atmosphere. For surfactant-assisted synthesis, it is difficult to remove the surfactants without compromising the mesoporosity. The larger the pores, the more readily the frameworks collapse.

There are so many living organisms with exquisite hierarchical structure and fantastic functions in nature, such as plant stems (Bae et al., 2014), butterfly wings (Peng et al., 2011), lotus leaves (Ensikat et al., 2011), and bone (Meyers et al., 2008), which exhibit highly organized hierarchical structures from the nano- to the micrometer scales, providing us a vivid inspiration and reference. We introduce the bionics ideas into the traditional preparation methods, imitating the hierarchical structure of biological systems to build hierarchical MOF structures and introducing the fine hierarchical structure of natural biomaterials into the MOFs to prepare a new type of hierarchical MOF materials. It is rare to introduce macro- and mesoporous structures into the MOFs at the same time to construct highly organized hierarchical structures, which are similar to those of the living organisms. The new hierarchical MOF materials therefore have highly ordered morphological structures and multilevel distribution in the continuous scale range. The ordered meso- and macrochannels can facilitate accessibility and mass transfer with high efficiency and can be applied in many fields.

As a major greenhouse gas, the continuous increase of carbon dioxide (CO_2_) in the atmosphere has caused serious environmental problems (Liao et al., 2015). However, CO_2_ is also an abundant, inexpensive, and nontoxic carbon source. However, due to its thermodynamic and kinetic stability, CO_2_ has been largely limited in chemical synthesis. Herein, we first synthesized the highly ordered macroporous-mesoporous MOFs, novel hierarchical MOF materials with three-scale porous sizes, the macroporous, mesoporous, and microporous structures, in one framework. Furthermore, both the macro- and mesoporous structures are highly ordered. Meanwhile, the ordered macroporous-mesoporous MOFs possess significant capture and selectivity for CO_2_ and can be used to catalyze the reaction of CO_2_ with benzyl halogen for chemical CO_2_ fixation under ambient conditions. These results provide a promising route for chemical CO_2_ fixation through MOF materials.

## Results and Discussion

**Figure 1:**
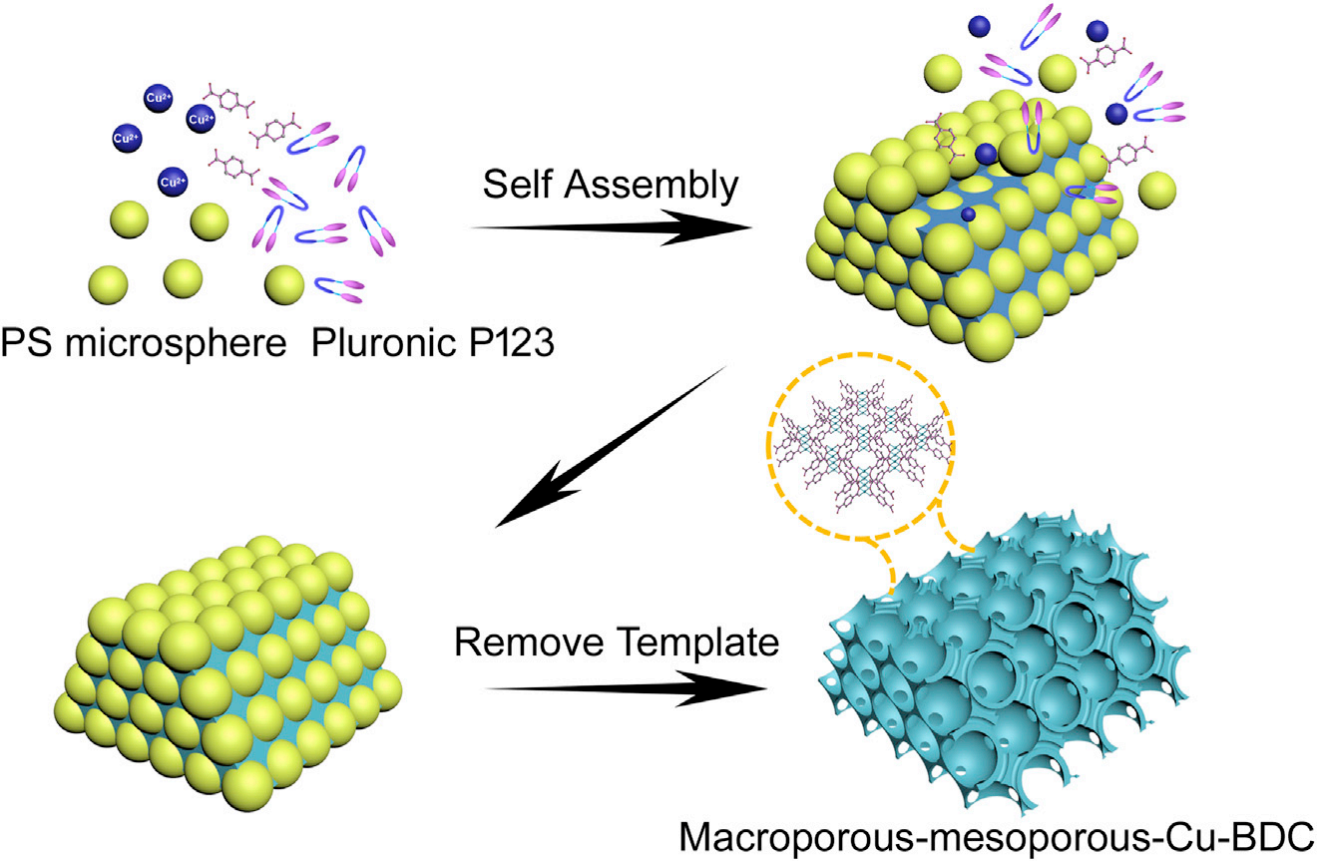
Schematic Illustration Schematic illustration of the synthesis procedure of the highly ordered macroporous-mesoporous MOFs.

We demonstrate a solvent evaporation-induced and co-assembly route to synthesize highly ordered macroporous-mesoporous MOFs with hierarchical structure using PS as the macroporous template and block copolymers as the mesoporous template (Figure 1). Both the macro- and mesoporous templates were assembled into the MOFs at the same time by ethanol evaporation process. After removing the templates, the highly ordered macroporous and mesoporous structures could be successfully introduced into the MOF structure to construct a hierarchical structure by using the one-pot method.

**Figure 2:**
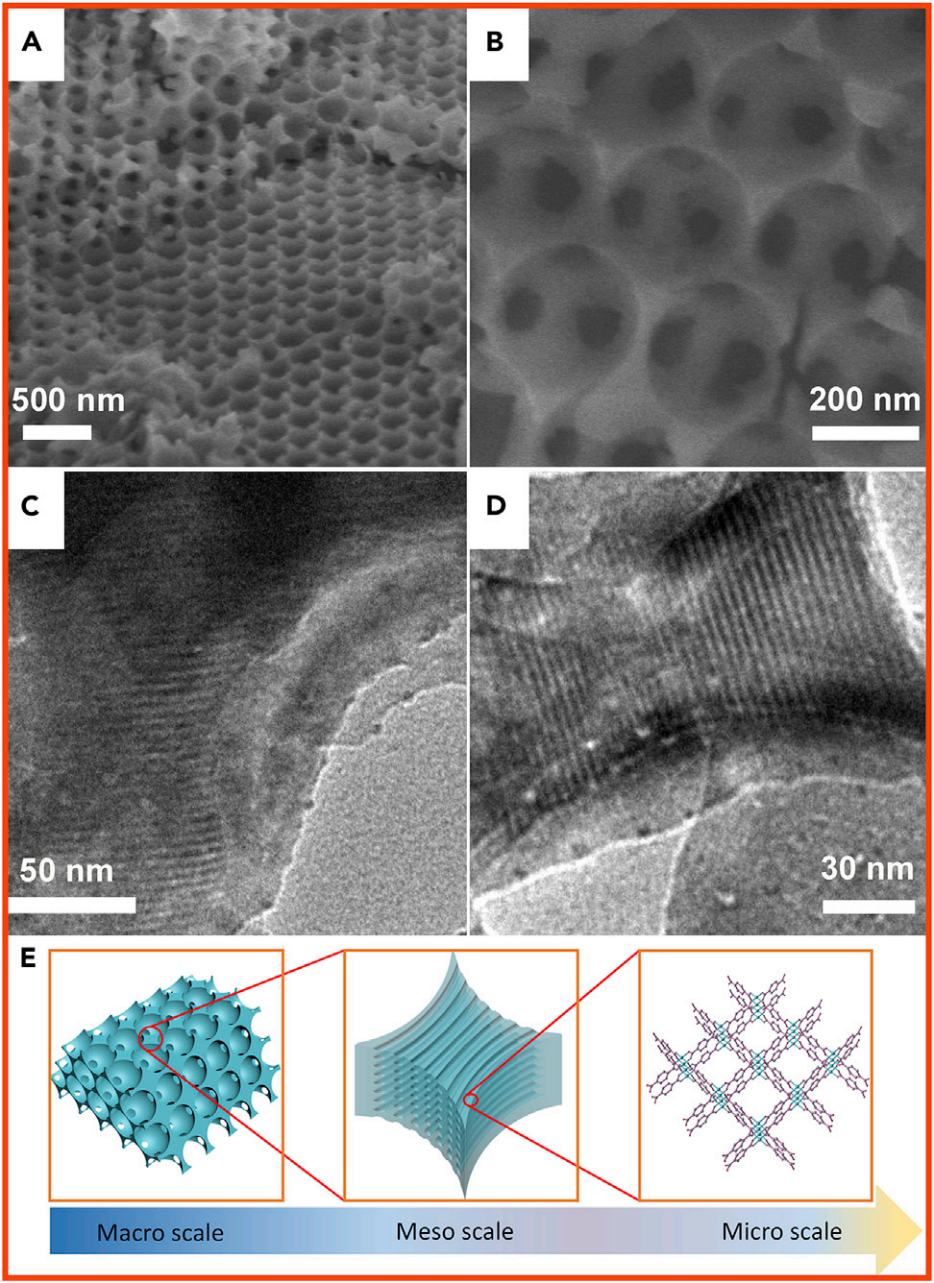
Pore Structure Characterization of Macro-Meso-Cu-BDC (A–D) (A) and (B) Scanning electron microscopic images and (C) and (D) TEM images of macro-meso-Cu-BDC. (E) Schematic illustration of hierarchical structure.

The hard template PS microspheres were uniform with diameters of 455 nm, as can be seen in Figure S2. Figures 2A and 2B show the representative scanning electron microscopic images of the synthesized macroporous-mesoporous MOFs (macro-meso-Cu-BDC), wherein the uniform macropores with ordered arrangement can be observed clearly. The well-ordered close-packed structures were organized into a typical face-centered cubic close-packed arrangement ordered over a range of tens of micrometers. The diameter of the macropores was 265 nm, which was smaller than that of the PS microspheres due to shrinkage during dissolution of the PS microspheres in organic solvents. The hexagonal mesoporous structure was confirmed by transmission electron microscopy (TEM). The TEM images (Figures 2C and 2D) show a high degree of periodicity in the mesoporous structure of the macroporous walls, which were in the [110] orientation, and the diameter of the uniform mesopores was 4.1 nm. For clearly conveying the idea here, Figure 2E shows the schematic illustration of the hierarchical structure.

**Figure 3:**
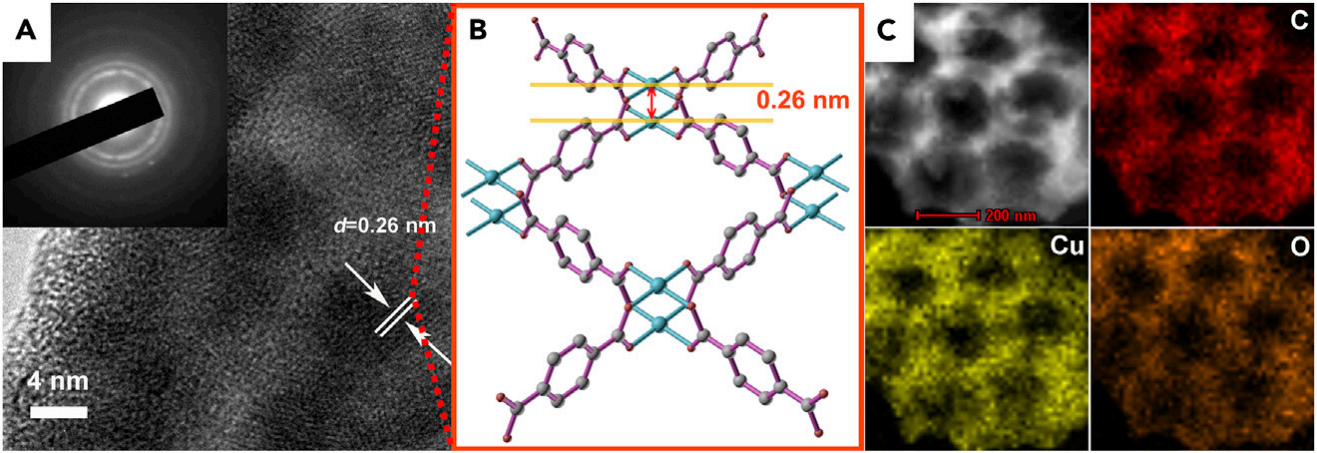
High-Resolution TEM, Energy-Dispersive X-Ray Spectroscopic Characterization, and Topology of Macro-Meso-Cu-BDC (A) High-resolution TEM image of macro-meso-Cu-BDC (the inset in (A) is the corresponding SAED pattern). (B) Cu-BDC topology. (C) Energy-dispersive X-ray spectroscopic element mappings of macro-meso-Cu-BDC.

The crystal lattice fringes were observed in high-resolution TEM image (Figure 3A), confirming the high crystallinity of the mesoporous walls. Lattice fringes with interplanar distance of 0.26 nm were observed, corresponding to the (001) plane of Cu-BDC. The crystalline nature of the mesoporous wall of the macro-meso-Cu-BDC was also verified by the observable polycrystalline diffraction rings in the selected area electron diffraction pattern (Figure 3A, inset). The elemental mapping image (Figure 3C) exhibits a homogeneous distribution of all the related elements, including C, O, and Cu, throughout whole macroporous-mesoporous MOFs.

**Figure 4:**
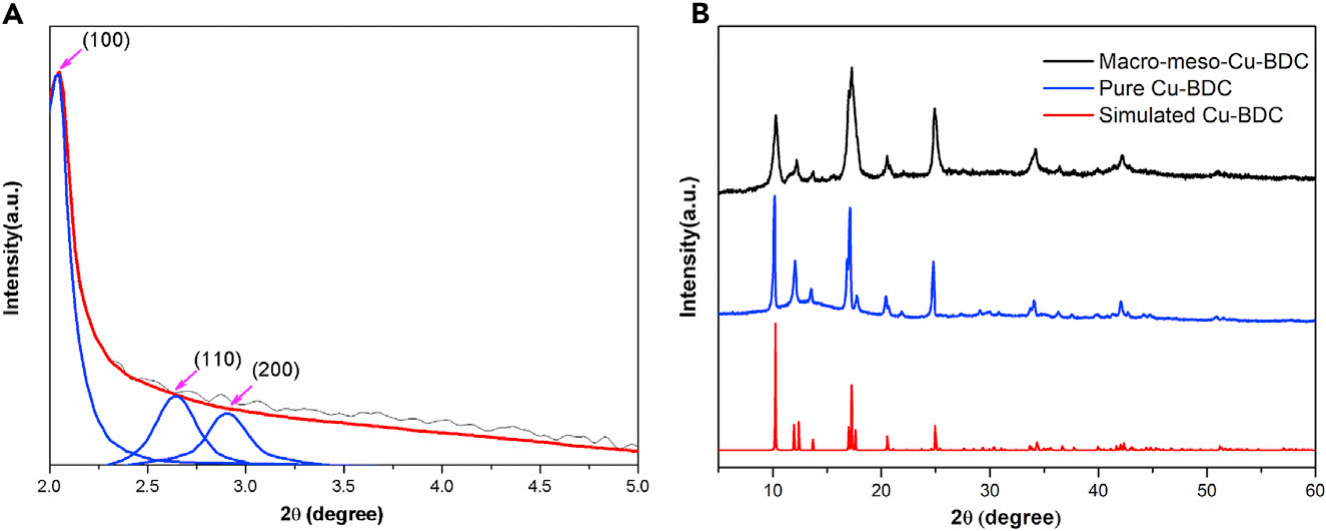
XRD Characterization of Macro-Meso-Cu-BDC (A) Small-angle X-ray diffraction pattern of macro-meso-Cu-BDC. (B) Wide-angle XRD patterns of macro-meso-Cu-BDC, when compared with pure Cu-BDC (blue line) and simulated Cu-BDC (red line).

The small-angle X-ray diffraction (XRD) pattern of the synthesized macro-meso-Cu-BDC (Figure 4A) demonstrates a typical hexagonal (*p6mm*) mesophase with a strong diffraction peak at 2θ = 2.04° and two small peaks at 2θ = 2.65° and 2.91°, which could be attributed to (100), (110), and (200) reflection, respectively. The corresponding *d* spacing is calculated to be 4.2 nm, which agrees well with the TEM result. Figure 4B shows wide-angle XRD patterns of the macro-meso-Cu-BDC. Compared with pure Cu-BDC (Carson et al., 2009) (blue line) and simulated Cu-BDC (red line), the reflection peaks of the macro-meso-Cu-BDC fit very well with pure Cu-BDC and simulated Cu-BDC, and no other peaks could be observed in the patterns, which demonstrates the formation of phase-pure Cu-BDC with highly crystalline mesopore walls. Furthermore, the XRD pattern of macro-meso-Cu-BDC shows broader diffraction peaks, suggesting that ordered mesostructure was obtained.

In agreement with XRD, the Fourier transform infrared (FTIR) spectra (Figure S3A) for macro-meso-Cu-BDC and pure Cu-BDC samples also represent the remaining unchanged main building block of the Cu-BDC structure. The appearance of a broad band at 3,000–3,700 cm^−1^ indicates the presence of -OH groups and water. The peaks at 1,576 cm^−1^ and 1,690 cm^−1^ correspond to the symmetric and asymmetric stretching vibrations of the carboxylate groups in Cu-BDC, respectively (Bhardwaj et al., 2015). In addition, the FTIR spectra for the sample without template removal, the sample with removal of only PS, and the sample with removal of only P123 were measured and demonstrated in Figure S4. The characteristic peak attributable to PS at 2,800–3,100 cm^−1^ disappeared, substantiating the fact that the PS template is all removed.

The thermal stabilities of the as-prepared macro-meso-Cu-BDC and pure Cu-BDC were examined by thermogravimetric analysis (TGA) under argon ambience. For the macro-meso-Cu-BDC (Figure S3B), the thermogravimetric profile can be divided into two steps: the vaporization of adsorbed water in the pores occurred at 130°C and then a sharp weight loss can be observed at temperatures between 250 and 440°C, which reflects the collapse of the framework due to the decomposition of the organic ligand. In the TGA profile of pure Cu-BDC, two obvious weight loss steps in the temperature range of 190°C–255°C and 325°C–460°C corresponding to the removal of the free molecules and the decomposition of the framework, respectively, were respectively found. The weight loss of macro-meso-Cu-BDC at lower temperatures than pure Cu-BDC further confirms the open meso- and macroporous hierarchical structure in the macro-meso-Cu-BDC.

**Figure 5:**
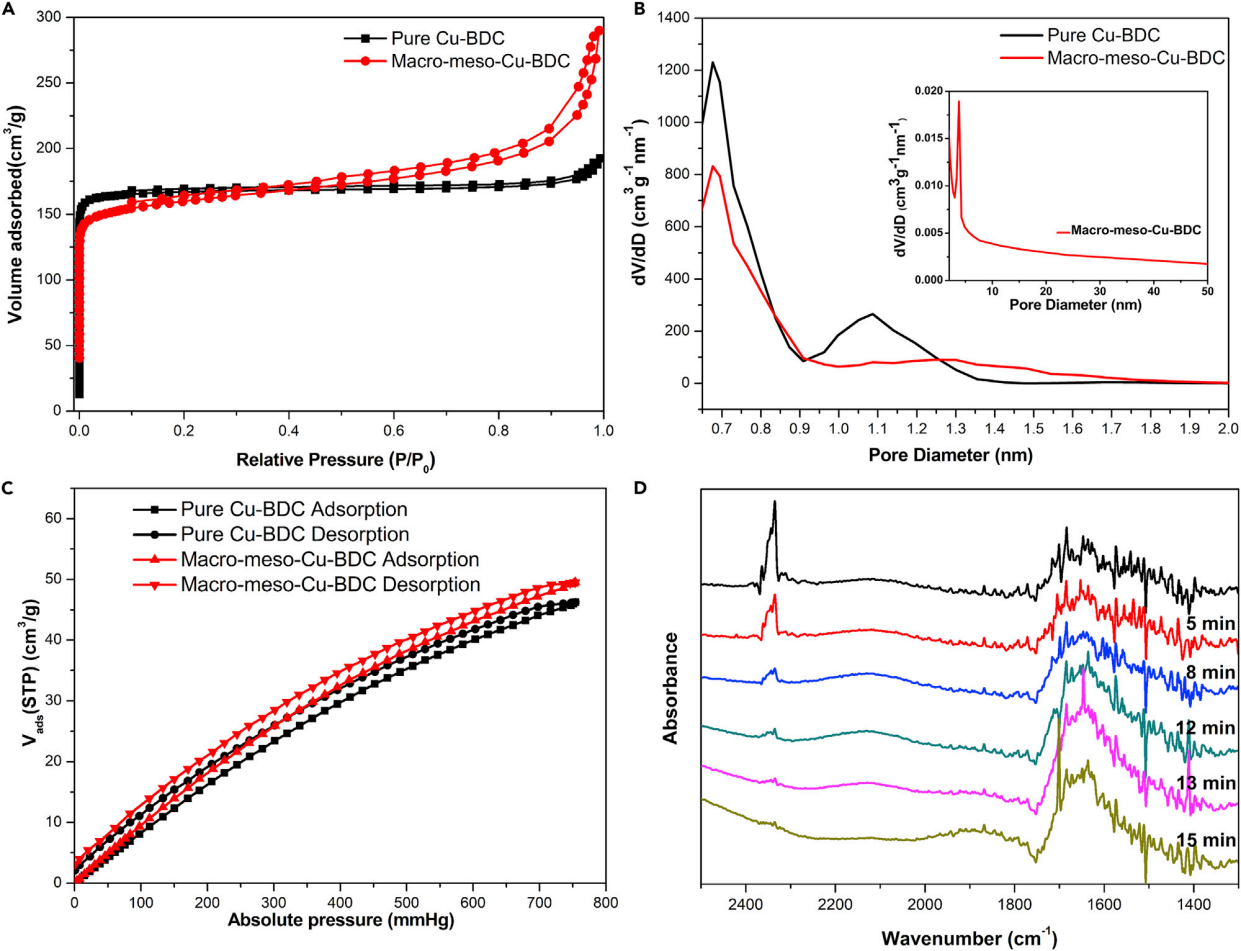
Characterization of Adsorption Capacity of Macro-Meso-Cu-BDC (A and B) (A) N_2_ adsorption isotherms and (B) pore size distribution analyses based on the nonlocal density functional theory method for macro-meso-Cu-BDC and pure Cu-BDC (the inset in (B) is the corresponding Barrett-Joyner-Halenda pattern). (C) CO_2_ adsorption isotherms of macro-meso-Cu-BDC and pure-Cu-BDC at 273 K. (D) DRIFT spectrum of CO_2_ adsorbed on macro-meso-Cu-BDC at room temperature (black line). Spectra collected with Ar purge for different time lengths: 5, 8, 12, 13, and 15 min.

To obtain further structural information, the porosities of pure Cu-BDC and macro-meso-Cu-BDC samples were measured by N_2_ adsorption-desorption isotherms at 77 K, the results of which are shown in Figure 5A. Pure Cu-BDC shows type I isotherms according to the International Union of Pure and Applied Chemistry classification (Sing, 1985), indicating the formation of microporous structures. Compared with pure Cu-BDC, the adsorption isotherms of macro-meso-Cu-BDC exhibited an intermediate mode between type I, characteristic of microporous structure, and type IV, characteristic of mesoporous structure, with a high nitrogen adsorption capacity at very low relative pressures. The hysteresis loop was observed to indicate the existence of mesoporous structure. Furthermore, the corresponding micropore size distribution curves of macro-meso-Cu-BDC and pure Cu-BDC were calculated using nonlocal density functional theory modeling method, and a narrow peak at 0.67 nm was observed in both the curves. Moreover, as calculated from the Barrett-Joyner-Halenda method (Figure 5B, inset), a uniform mesopore size of 3.9 nm was obtained in the macro-meso-Cu-BDC, which agrees well with the TEM result. The Brunauer-Emmett-Teller surface area of macro-meso-Cu-BDC was 592 m^2^/g, which was higher than that of the pure Cu-BDC (540 m^2^/g). It suggests that the macro- and mesoporous structures are in favor of the improvement of the surface areas in the macro-meso-Cu-BDC. The CO_2_ uptake isotherms of the samples measured at 273 K and up to 1 bar are shown in Figure 5C. The macro-meso-Cu-BDC exhibited a high CO_2_ adsorption capacity with 49.51 cm^3^/g, whereas pure Cu-BDC gave a lower CO_2_ capacity with only 46.23 cm^3^/g. It suggests that CO_2_ adsorption capacity of the macro-meso-Cu-BDC is higher than that of pure Cu-BDC, which is related to the hierarchical structures of macro-meso-Cu-BDC. The large CO_2_ adsorption capacity of macro-meso-Cu-BDC may be attributed to its high specific surface area compared with that of pure Cu-BDC.

To further investigate the interaction between macro-meso-Cu-BDC and CO_2_, *in situ* diffuse reflectance infrared Fourier transform (DRIFT) experiments were carried out at room temperature (Figure 5D). During the CO_2_ adsorption, the macro-meso-Cu-BDC displayed a distinct and intense band centered at approximately 2,335 cm^−1^ corresponding to the ν3 mode of CO_2_, which showed a significant red shift compared with that of the gas phase (2,349 cm^−1^). It is due to the formation of the H-bonds between CO_2_ and the -OH groups of Cu-BDC (Vimont et al., 2007, Lan et al., 2011). There were also several peaks discovered in the range of 1,800 to 1,300 cm^−1^, which originated from the interaction between CO_2_ and -OH groups (Ferretto and Glisenti, 2003). After stopping CO_2_ supply, the DRIFT spectra were collected under argon purging. It was found that the characteristic band at 2,335 cm^−1^ significantly decreased with time and almost disappeared from the spectra after 15-min purging. However, the intensities of peaks at 1,800 to 1,300 cm^−1^ remain unchanged, disclosing a strong bonding between CO_2_ and the macro-meso-Cu-BDC.

In addition, the basicity of the as-synthesized macro-meso-Cu-BDC and the corresponding pure Cu-BDC were also evaluated by CO_2_-temperature-programmed reduction (TPD) experiments in Figure S5. CO_2_ was adsorbed at 25°C and then desorbed in the range of 25°C–700°C. The macro-meso-Cu-BDC and the pure Cu-BDC both showed two obvious CO_2_ desorption peaks around 372°C and 424°C and 400°C and 436°C, respectively. It indicated the presence of different surface basic sites. Based on the peak area, higher values of basicity were observed for the macro-meso-Cu-BDC than those for the pure Cu-BDC, indicating a larger CO_2_ adsorption capacity and more active sites for CO_2_.

The high CO_2_ adsorption capacity and the unique hierarchical multiscale porous framework indicate that the macro-meso-Cu-BDC can be a highly promising heterogeneous adsorbent and catalyst for the chemical fixation of CO_2_. The CO_2_ carbonylative coupling reaction with 4-methylbenzyl chloride was first chosen to evaluate the performance of macro-meso-Cu-BDC at relatively mild conditions of 0.1 MPa CO_2_ and 100°C; the desired carbonylative coupling product **1** was obtained with yield of 68%, accompanied by the by-product **2** in 3% yield, which reacted with *N*,*N*-Dimethylformamide (DMF) (Table S1, entry 1). Unlike the macro-meso-Cu-BDC-catalyzed reaction, when pure Cu-BDC was used as the catalyst, the reaction showed a lower yield and selectivity and only a small amount (8%) of **1** was afforded, with **2** in 10% yield (Table S1, entry 2). At the same time, the meso-Cu-BDC has only 13% yield and 6% selectivity (Table S1, entry 3). Comparing various copper salts, the results show that the copper salt does not have any effect on the reaction (Figure S7, Table S1, entries 4–7). Macro-meso-Cu-BDC showed an excellent catalytic performance in the chemical fixation CO_2_ reaction, which could significantly enhance the yield of the desired product, because ordered meso- and macrochannels existed in the MOFs, which can facilitate accessibility and mass transfer with high efficiency. Owing to the unique hierarchical pores, high surface area, and high CO_2_ adsorption capacity, macro-meso-Cu-BDC exposes more active sites, which is more conducive to adsorbing and activating large amounts of CO_2_ to catalyze the reaction at copper active sites.

To demonstrate the scope and applicability of this macro-meso-Cu-BDC for other structurally diverse benzyl halogen, the CO_2_ carbonylative coupling reactions with various benzyl halogens are carried out, and the results were summarized in Table 1. The reactions of 4-nitrobenzyl chloride (**1a**), 4-fluorobenzyl chloride (**1b**), and 4-methoxybenzyl chloride (**1c**) that bear strong electron-withdrawing groups on the *para*-position provide high yields for products **2a**, **2b**, and **2c** under the same reaction conditions (entries 1–3, 77%, 71%, and 70%). By only reducing the amount of Cs_2_CO_3_, products **2d** and **2e** were also obtained in good yield from the reactions of benzyl chlorides **1d** and **1e** bearing an electron-donating group methyl (Me) on the *para*-position (entry 4, 68%) and *meta*- position (entry 5, 66%). Furthermore, 4-tert-butylbenzyl chloride (**1f**) was converted with an yield of 50% (entry 6). Moreover, benzyl chloride (**1g**) and benzyl bromide (**1h**) were also utilized in this type of carboxylative coupling reaction. Products **2g** and **2h** are obtained with yields of 60% and 43%, respectively (entries 7–8). Therefore this catalysis system was quite versatile, as a variety of benzyl halogens could be converted to the corresponding phenyl ester in excellent yields. The XRD pattern and TEM image of macro-meso-Cu-BDC after catalytic reaction showed that the phase and pore structures of the catalyst remained almost unchanged, which also confirmed the stability of the catalyst (Figure S8).

**Table 1:** Summary of the Results of CO_2_ Carbonylation Coupling Reactions

a			
Entry	Benzyl Halogen	Product	Yield (%)^b^
1	**1a**	**2a**	77
2	**1b**	**2b**	71
3	**1c**	**2c**	70
4^c^	**1d**	**2d**	68
5^c^	**1e**	**2e**	66
6^c^	**1f**	**2f**	50
7^c^	**1g**	**2g**	60
8^c^	**1h**	**2h**	43

CO_2_ fixation with benzyl halogen with different functional groups catalyzed by macro-meso-Cu-BDC.^a^

a Reaction conditions: benzyl halogen (2 mmol), NaBH_4_ (2 mmol), Cs_2_CO_3_, (0.4 mmol), CO_2_ (0.1 MPa), catalyst (10 mol %), DMF (8 mL) at 100°C for 8 h.

b Isolated yield.

c Cs_2_CO_3_, (0.2 mmol).

### Conclusion

In summary, the novel highly ordered hierarchical macro-meso-Cu-BDC with crystalline mesoporous walls was successfully synthesized by using a facile solvent evaporation induced co-assembly route in one step. These hierarchical MOF materials with three-scale porous sizes, which are the macro-, meso-, and microporous structures, have been presented in one framework. The highly ordered meso- and macrochannels can further improve the mass transfer efficiency. Owing to the unique hierarchical pores, high surface area, and high CO_2_ adsorption capacity, the macro-meso-Cu-BDC possesses high activity for chemical fixation of CO_2_ under mild conditions. These results provide a promising route to chemical CO_2_ fixation through MOF materials.

### Limitations of Study

Owing to the poor structural stability of macro-meso-Cu-BDC, we do not know if it can be used for longer period (>50 cycles) catalytic reactions.

## Methods

All methods can be found in the accompanying Transparent Methods supplemental file.

## Supplemental Information

Document S1. Transparent Methods, Figures S1–S8, and Table S1
