# Supplementary material for: Hierarchical Structure with Highly Ordered Macroporous-Mesoporous Metal-Organic Frameworks as Dual Function for CO2 Fixation
Source: iScience. 2019 May 9;15:514–23. doi: 10.1016/j.isci.2019.05.006 (PMC6538925; doi:10.1016/j.isci.2019.05.006)

ISCI, Volume *15*

## Supplemental Information

# Hierarchical Structure with Highly Ordered Macroporous-Mesoporous Metal-Organic Frameworks as Dual Function for $CO_2$ Fixation

**Zhenxing Li, Xiaofei Xing, Dong Meng, Zhengxu Wang, Jingjing Xue, Rui Wang, Junmei Chu, Mingming Li, and Yang Yang**

# Supporting Information

## Hierarchical Structure with Highly Ordered Macroporous-Mesoporous MOFs as Dual-Function for $CO_2$ Fixation

*Zhenxing Li,* Xiaofei Xing, Dong Meng, Zhengxu Wang, Jingjing Xue, Rui Wang, Junmei Chu, and Mingming Li, and Yang Yang**

### Transparent Methods

*Synthesis of PS microspheres 455*

In a typical synthesis, styrene was washed with NaOH solution and distilled water until the pH value is neutral. 12 ml of styrene and 160 ml of deionized water were added into a 250 ml three-necked round-bottom flask, and nitrogen was bubbled for 15 minutes to remove the oxygen. And then the solution was heated to 70 °C and 10 mL of $K_2S_2O_8$ (0.007 g· $mL^{-1}$) was added. The reaction was continued for 28 hours under nitrogen protection. The obtained mixture was washed with deionized water and ethanol for three times. Finally, white powder PS microspheres were obtained after dried in air at 30 °C.

*Synthesis of macro-meso-Cu-BDC*

In a typical synthesis, 1.5 g of P123 was dissolved in 30 ml of ethanol, after P123 was completely dissolved, 1.0 g of PS microspheres (d = 455 nm) were added and continued stirring for another 4 hours to ensure the complete dispersion of the PS microspheres. Then 0.121 g of Cu $(NO_3)_2$•$3H_2O$ was added to the above solution, and after stirring for 2 hours, 0.083 g of p-phthalic acid was added. After continuing stirring for a further 3 hours at room temperature, the solution was transferred to an oven at 30 °C for 5 days. After removal from the oven, washing several times with DMF (elimination of ligand and PS microspheres). The template P123 was then removed using an acidic ethanol solution (the volume ratio of acetic acid and ethanol=1:50). At last, the above solution was placed in a 60 °C vacuum oven for 10 hours to obtain the blue solid powder.

*Synthesis of meso-Cu-BDC*

In a typical synthesis, 1.5 g of P123 was dissolved in 30 ml of ethanol, after P123 was completely dissolved. Then 0.121 g of Cu $(NO_3)_2{\bullet}3H_2O$ was added to the above solution, and after stirring for 2 hours, 0.083 g of p-phthalic acid was added. After continuing stirring for a further 3 hours at room temperature, the solution was transferred to an oven at 30 °C for 5 days. The template P123 was then removed using an acidic ethanol solution (the volume ratio of acetic acid and ethanol=1:50). At last, the above solution was placed in a 60 °C vacuum oven for 10 hours to obtain the blue solid powder.

*Synthesis of pure Cu-BDC.*

0.530 g of $Cu(NO_3)_2{\bullet}6H_2O$ was dissolved in 45 ml of DMF, after stirring for 2 hours, 0.362 g of p-phthalic acid was added and continued stirring for another 2 hours. Then the mixture was transferred to a reactor and reacted at 110 ℃ for 36 hours. After that, the mixture was washed with DMF and ethanol three times and dried at 40 °C for 8 hours in in vacuum to obtain the blue solid powder.

*Representative procedure for catalytic reactions*

In a typical experiment, the reaction was performed in a sealed Schlenk tube using benzyl halogen (0.2532 g, 2 mmol), $NaBH_4$ (0.0757 g, 1 eq.), $Cs_2CO_3$ (0.0652 g, 0.4 mmol) and macro-meso-Cu-BDC (10 mol %) in anhydrous DMF at 0.1 MPa and 100 °C for 4 h under the $CO_2$ atmosphere. The crude product was purified by column chromatography (petroleum ether: ethyl acetate=30:1) to give a white solid. The resulting products were characterized by $^1$HNMR.

*Characterization*

The scanning electron microscopy (SEM) observations were taken by Hitachi SU8010 scanning electron microscopy at an accelerating voltage of 30 kV. Transmission electron microscopy (TEM) and HRTEM images were carried out on a JEM 2100 LaB6 at 200 kV. The energy-dispersive X-ray analysis (EDS) were recorded on a Hitachi SU8010 scanning electron microscopy under a working voltage of 30 kV. The X-ray diffraction (XRD) data were carried out on a Burker D8-advance X-ray power diffractometer with Cu-Kα radiation (α=1.5406 Å). The FT-IR spectra were determined at room temperature on a Perkin Elmer Frontier spectrometer (equipped with a DTGS detector). Thermal gravimetric analyses (TGA) were recorded on Mettler Toledo TGA/DSC 3+ thermogravimetric

analyzer under argon atmosphere at a heating rate of 10 °C $min^{-1}$ in the temperature range of 100-700 °C. $N_2$ adsorption-desorption isotherms were obtained on a Micromeritics ASAP 2460 analyzer (USA) at liquid nitrogen temperature (77 K). The samples were degassed in a vacuum at 80 °C for 6 h prior to the measurement. Pore size distributions were estimated using non-local density functional theory (NLDFT) and BJH (Barret-Joyner-Halenda) model. The surface areas were calculated by the BET (Brunauer-Emmett-Teller) method. The $CO_2$ sorption isotherms were measured by an automatic adsorption equipment (Autosorb-iQ-MP). Liquid $^1$HNMR spectra were taken using $CDCl_3$ as the solvent at 600 MHz with a JNM-ECA600 spectrometer. The DRIFT spectra of $CO_2$ adsorption were performed on a Nicolet iS10 spectrometer with an MCT detector. The samples were activated at 160 °C for 12 h before characterization. After that, the samples purged by argon flow (20 mL/min) for 20 min at room temperature. Then the spectra of $CO_2$ adsorption were collected under a $CO_2$ flow (3 mL/min) and the weakly adsorbed $CO_2$ was removed under an Ar flow (5 mL/min). $CO_2$-TPD experiments were performed on a Quantachrome CHEMBET-3000 TPD instrument equipped with a TCD detector. The samples heated to 120 °C and held at this temperature for 30 min under a He flow (25 mL/ min) to eliminate water and adsorbed $CO_2$. Then, the temperature was reduced to 25 °C, and $CO_2$ flow (70 mL/ min) contacted the samples for 30 min. After that, the samples were submitted to a He flow (25 mL/ min) and the $CO_2$ desorption was carried out between 25 and 700 °C at a rate of 10 °C/min.

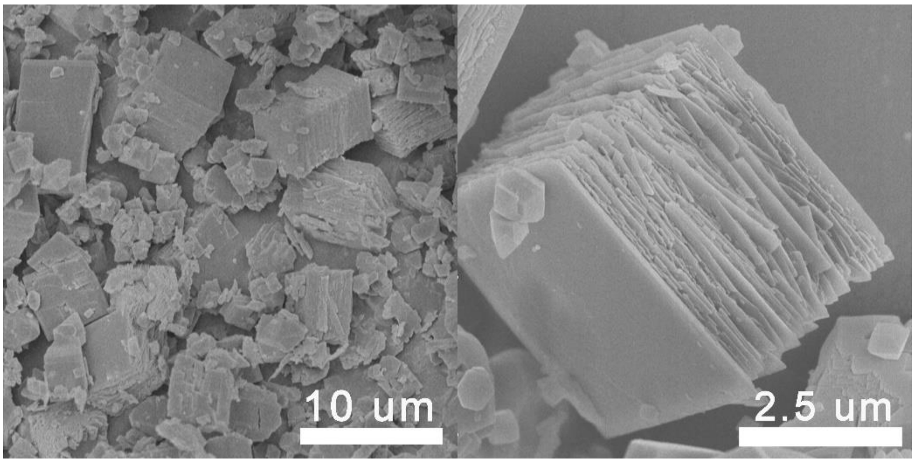


**Figure S1.** SEM image of pure Cu-BDC. (related to Figure 1)

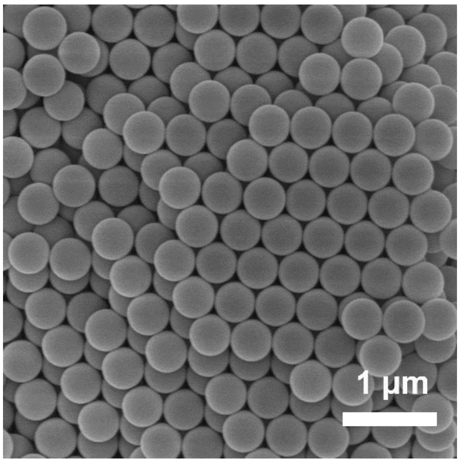


**Figure S2.** SEM image of the prepared PS microspheres with the diameter of 455 nm. (related to Figure 1)

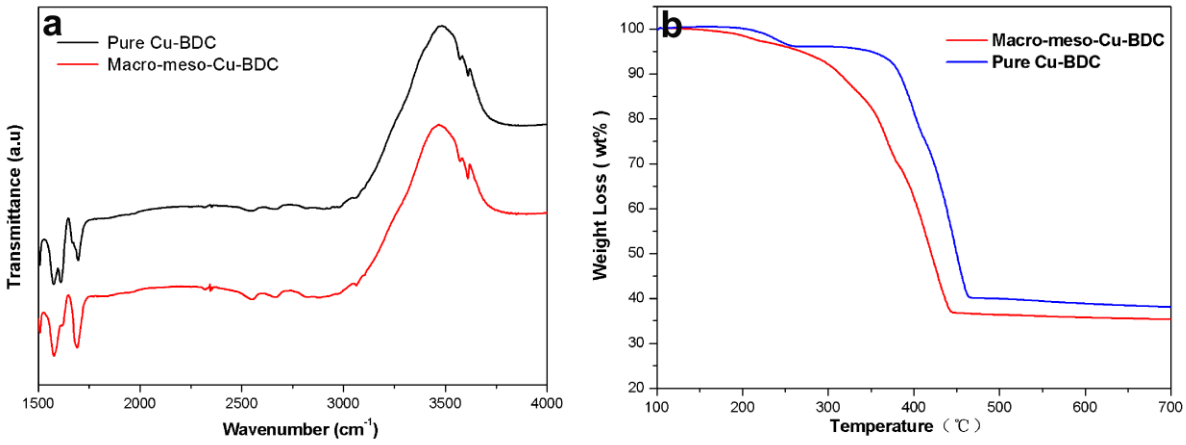


**Figure S3.** (a) FT-IR spectra of macro-meso-Cu-BDC and pure Cu-BDC, respectively. (b) Thermogravimetric analysis (TGA) of macro-meso-Cu-BDC and pure Cu-BDC in Ar atmosphere, respectively, with a heating rate of 5 °C per minute. (related to Figure 3)

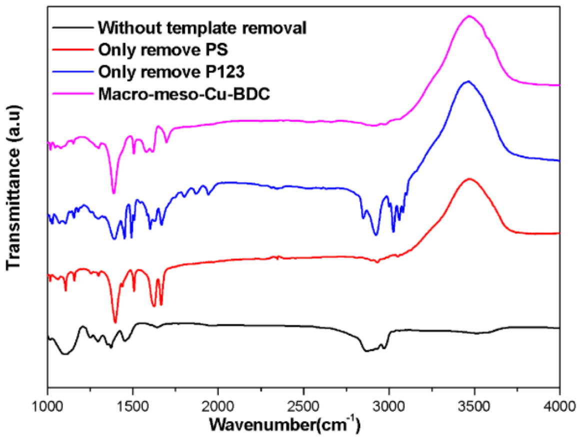


**Figure S4.** FT-IR spectra of macro-meso-Cu-BDC, the sample without template removal, the sample only remove of PS and the sample only remove of P123, respectively. (related to Figure 3)

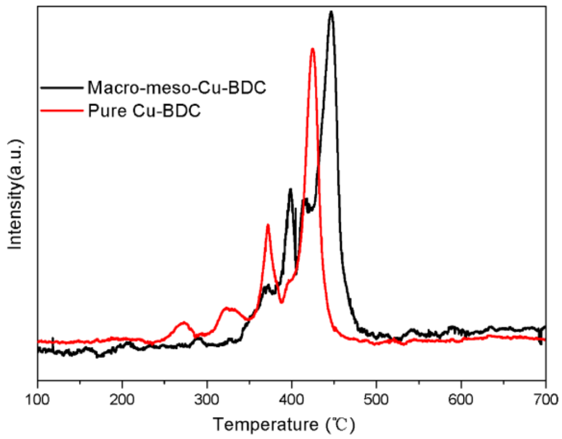


**Figure S5.** $CO_2$-TPD spectra of macro-meso-Cu-BDC and pure Cu-BDC. (related to Figure 5)

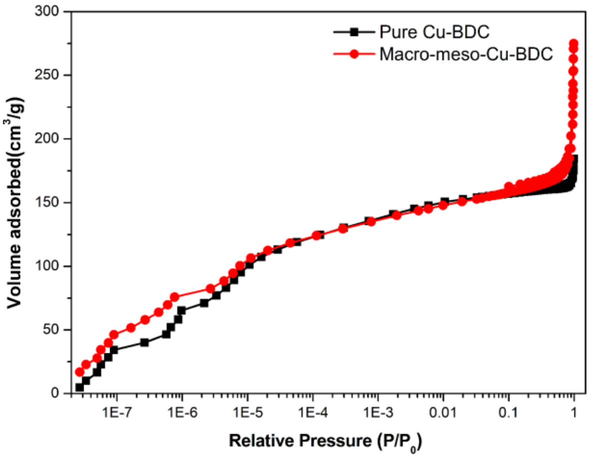


**Figure S6.** log-scale $N_2$ adsorption isotherms for macro-meso-Cu-BDC and pure Cu-BDC. (related to Figure 5)

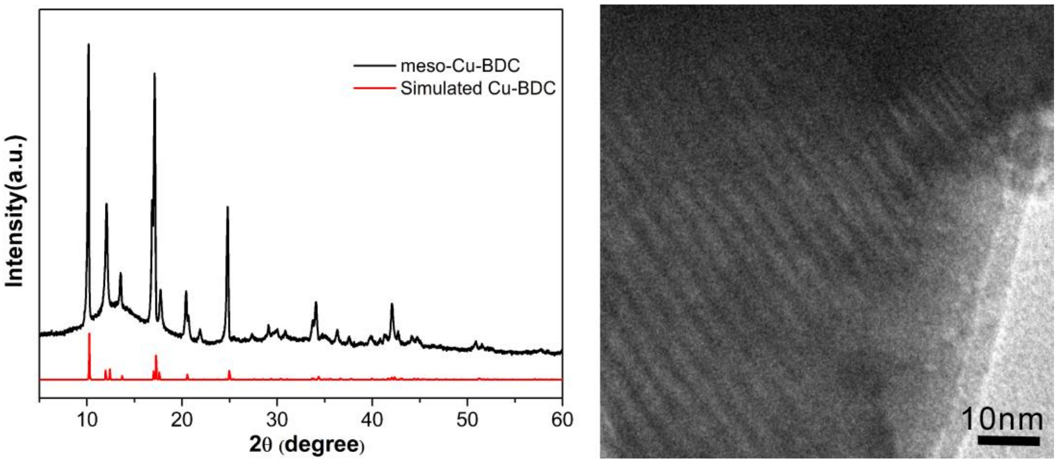


**Figure S7**. XRD pattern and TEM image of meso-Cu-BDC. (related to Table 1)

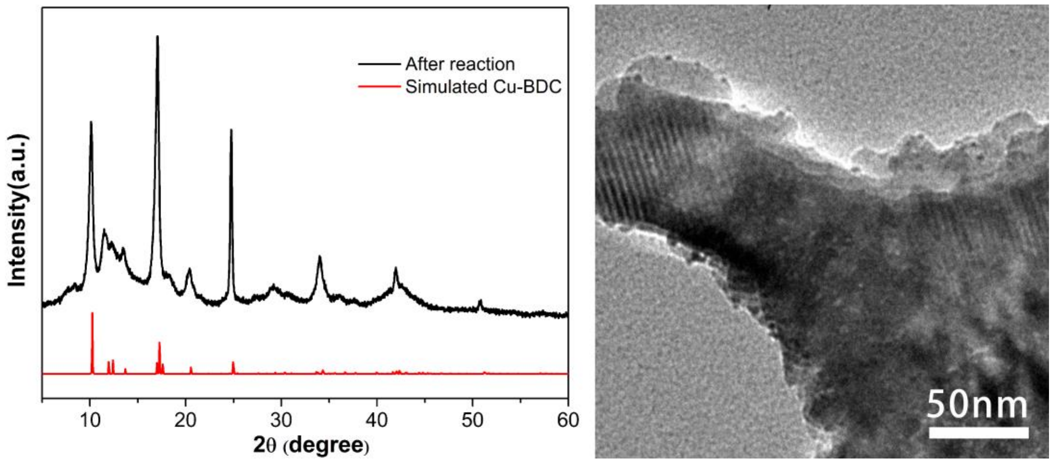


**Figure S8**. XRD pattern and TEM image of macro-meso-Cu-BDC after catalysis. (related to Table 1)

**Table S1.** $CO_2$ fixation with 4-methylbenzyl chloride catalyzed by macro-meso-Cu-BDC, pure Cu-BDC and meso-Cu-BDC, respectively.[a)] (related to Table 1)

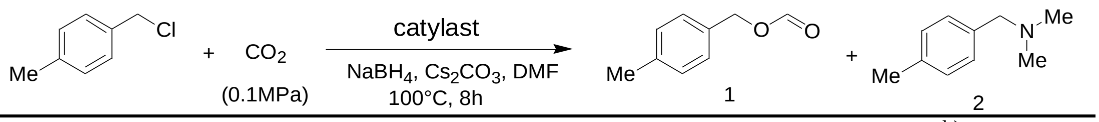


| Entry | 4-Methylbenzyl chloride | Catalyst | Yield (%)[b)] 1 | 2 |
|---|---|---|---|---|
| 1 | 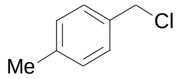 | Macro-meso-Cu-BDC | 68 | 3 |
| 2 | 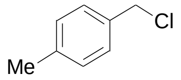 | Pure Cu-BDC | 8 | 10 |
| 3 | 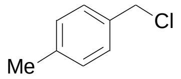 | Meso-Cu-BDC | 13 | 6 |
| 4 | 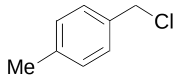 | $CuCl_2$ | Not Detected | Not Detected |
| 5 | 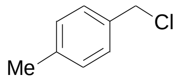 | $CuSO_4$ | Not Detected | Not Detected |
| 6 | 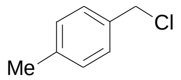 | $Cu(NO_3)_2{\cdot}3H_2O$ | Not Detected | Not Detected |
| 7 | 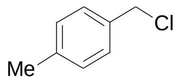 | $Cu(AC)_2{\cdot}H_2O$ | Not Detected | Not Detected |
| 8 [c)] | 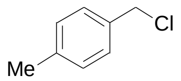 | Macro-meso-Cu-BDC | 66 | 4 |

[a]Reaction conditions: 4-methylbenzyl (2 mmol), $NaBH_4$ (2 mmol), $Cs_2CO_3$, (0.4 mmol), $CO_2$ (0.1 MPa), catalyst (10 mol %), DMF (8 mL) at 100 °C for 8 h. [b]Isolated yield. [c]Reaction conditions: 4-methylbenzyl (2 mmol), $NaBH_4$ (2 mmol), $CsF_3$, (0.4 mmol), $CO_2$ (0.1 MPa), catalyst (10 mol %), DMF (8 mL) at 100 °C for 8 h.

## $^1$HNMR (related to Table 1):
## Product 2a

$^1$HNMR (600 MHz, CHLOROFORM-D) δ 8.21 (d, J = 8.7 Hz, 3H), 7.53 (d, J = 8.8 Hz, 2H), 4.83 (s, 2H).

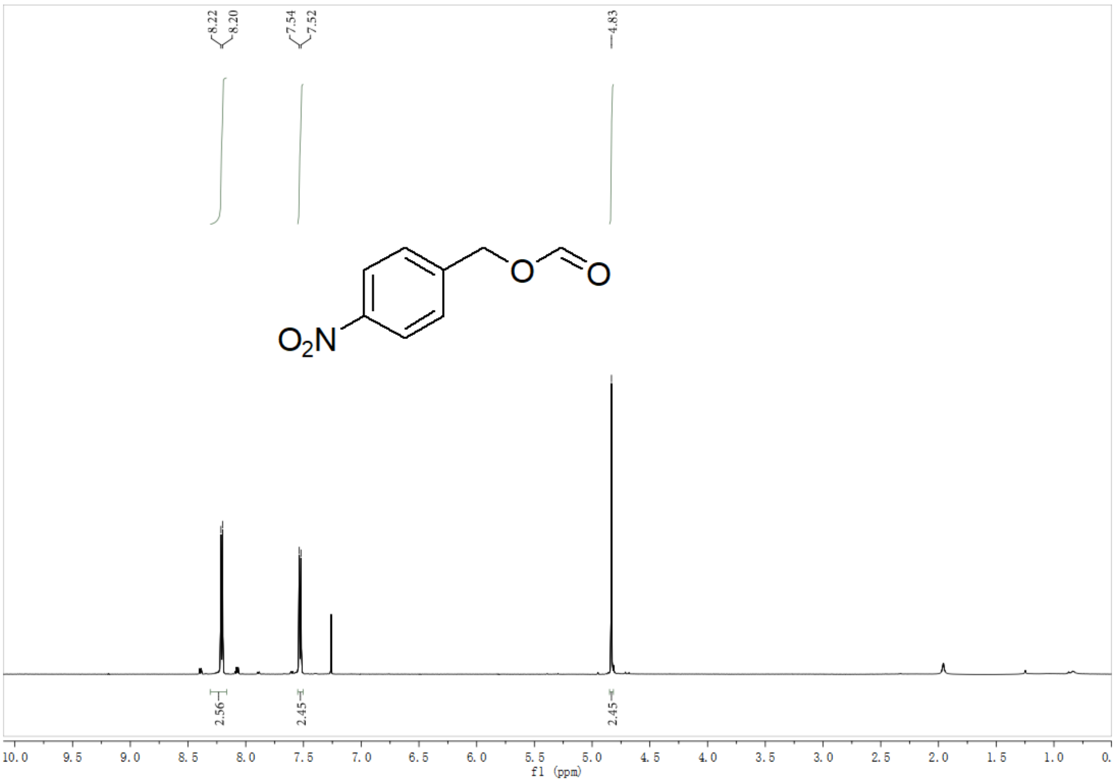

**Product 2b**

$^{1}$HNMR (600 MHz, CHLOROFORM-D) δ 8.11 (s, 1H), 7.43 (d, J = 8.5, 5.3 Hz, 2H), 7.08 (t, J = 8.7 Hz, 2H), 5.34 (s, 2H).

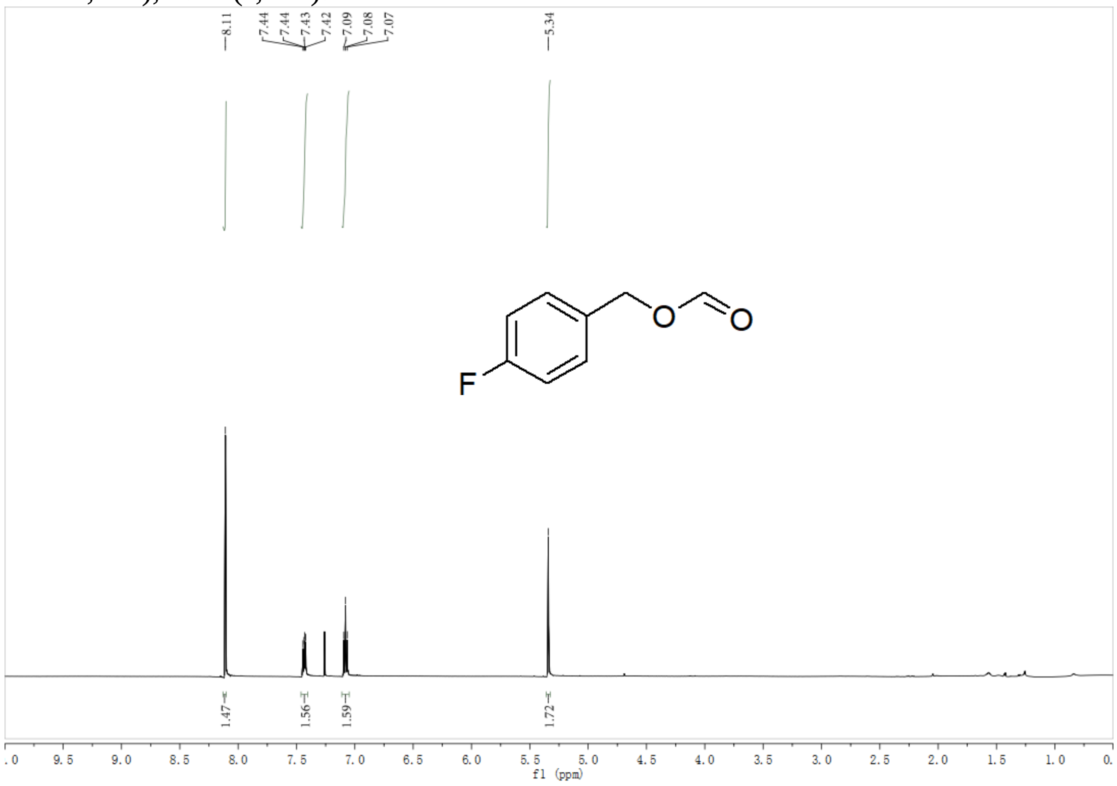


**Product 2c**

$^{1}$HNMR (600 MHz, CHLOROFORM-D) δ 8.09 (s, 1H), 7.39 (d, J = 8.4 Hz, 2H), 6.92 (d, J = 8.5 Hz, 2H), 5.31 (s, 2H), 3.82 (s, 3H).

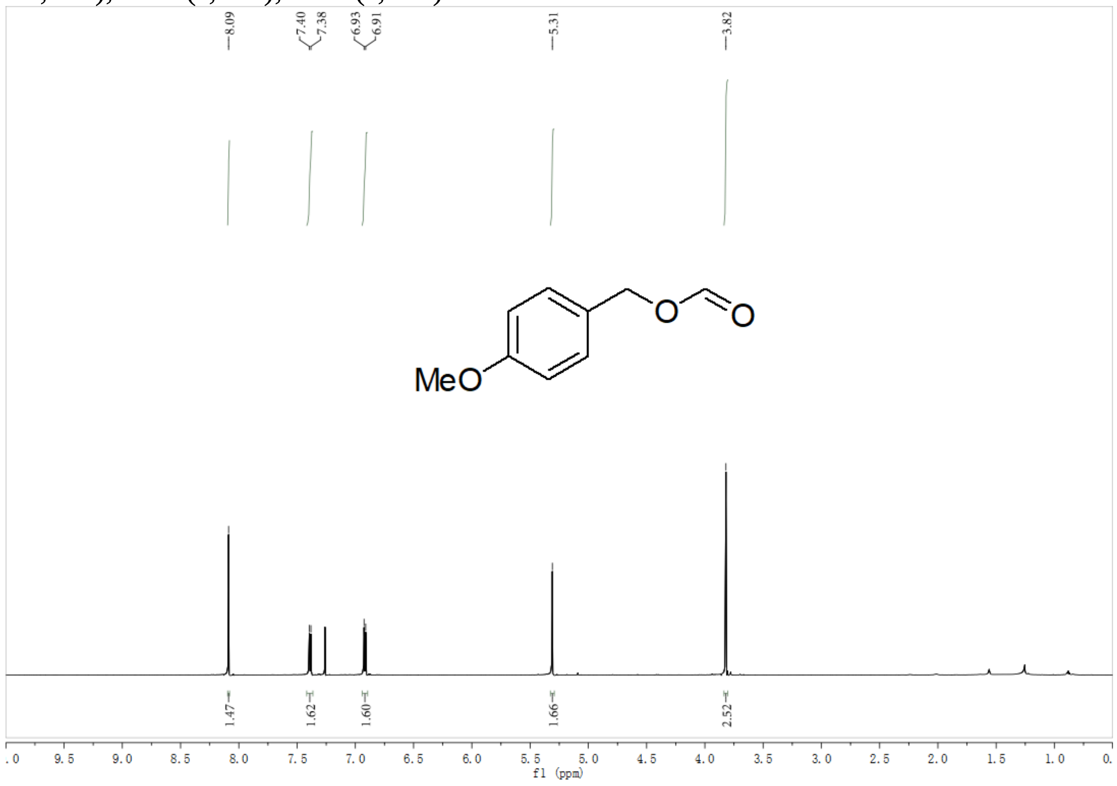

**Product 2d**

$^{1}$HNMR (600 MHz, CHLOROFORM-D) δ 8.11 (s, 1H), 7.35 (d, J = 7.9 Hz, 2H), 7.21 (d, J = 7.8 Hz, 2H), 5.34 (s, 2H), 2.37 (s, 3H).

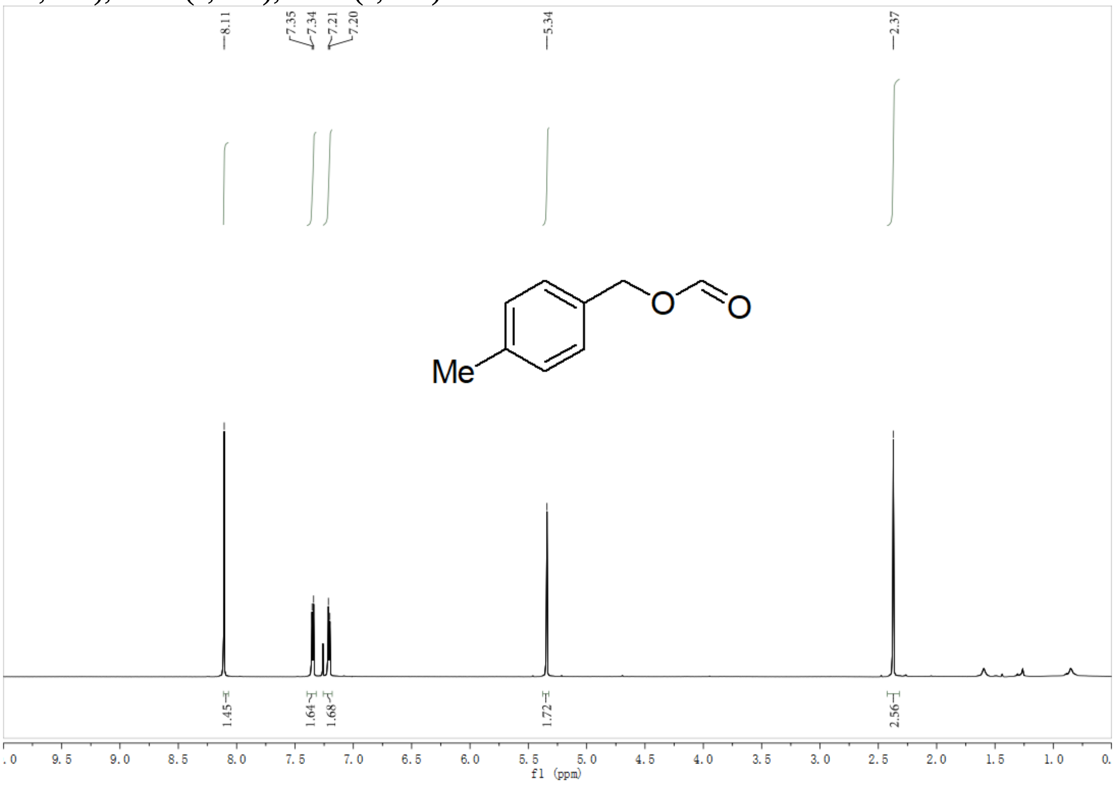


**Product 2e**

$^{1}$HNMR (600 MHz, CHLOROFORM-D) δ 8.13 (s, 1H), 7.25 (d, J = 48.7, 7.4 Hz, 3H), 7.16 – 7.10 (m, 1H), 5.35 (s, 2H), 2.38 (s, 3H).

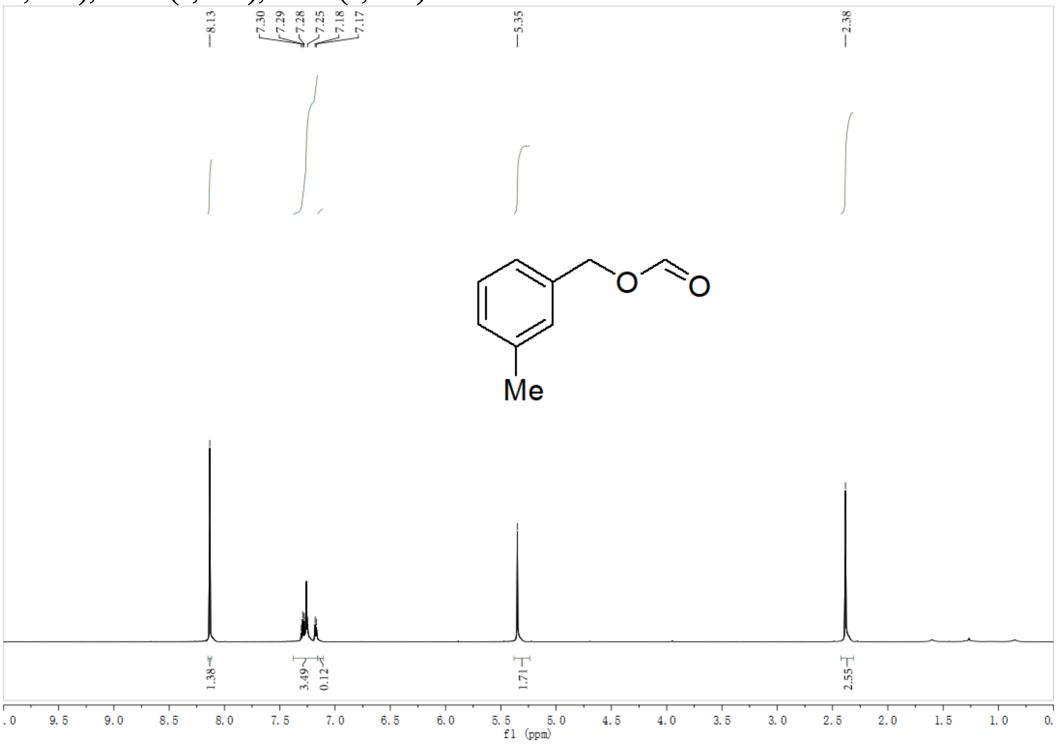

**Product 2f**

$^{1}$HNMR (600 MHz, CHLOROFORM-D) δ 8.12 (s, 1H), 7.41 (d, J = 21.0, 8.4 Hz, 4H), 5.35 (s, 2H), 1.33 (s, 9H).

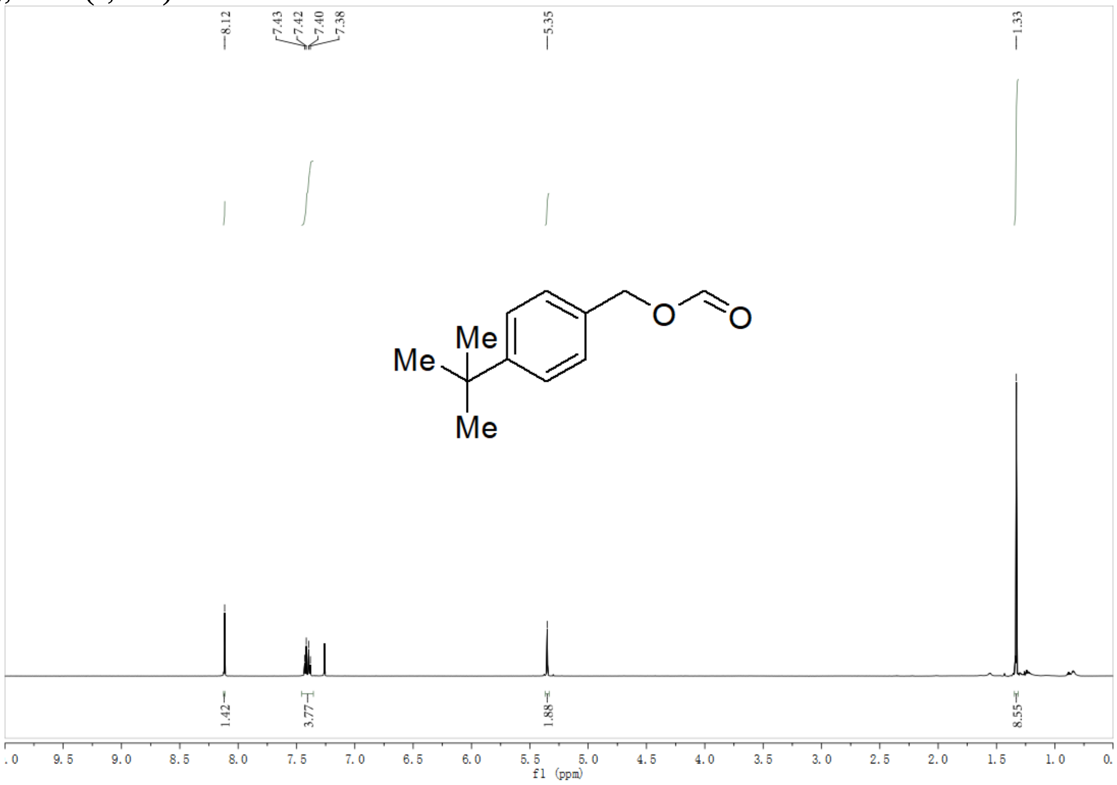


**Product 2g**

$^{1}$HNMR (600 MHz, CHLOROFORM-D) δ 8.13 (s, 1H), 7.40 (ddd, J = 25.2, 20.8, 7.2 Hz, 5H), 5.38 (s, 2H).

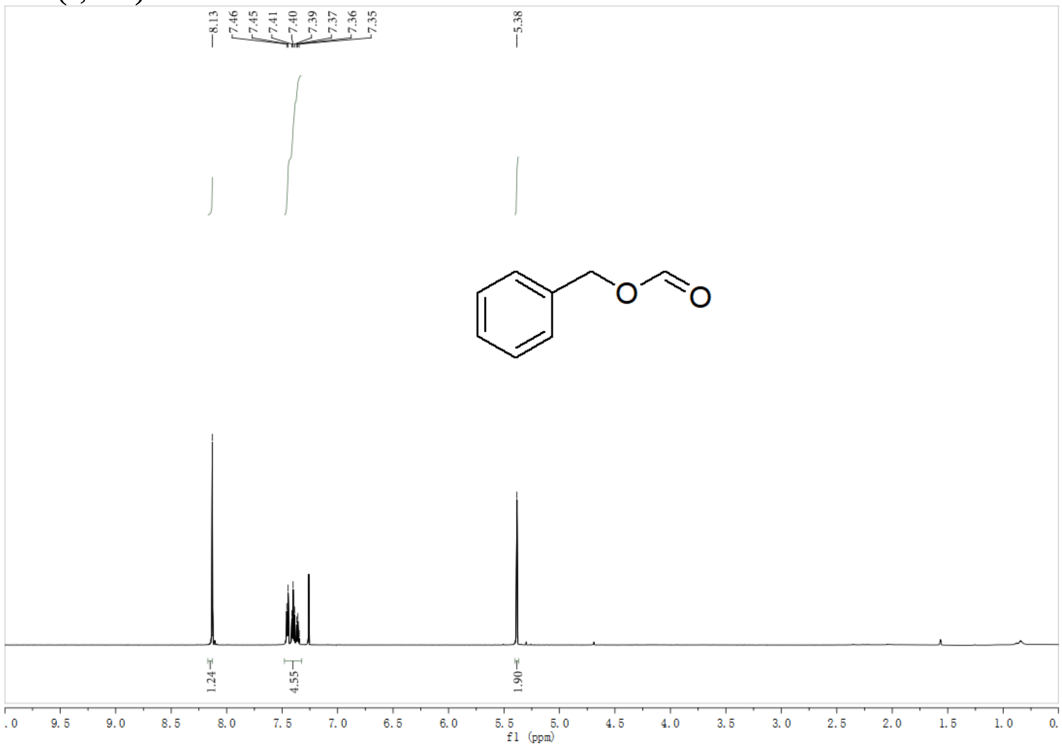

## Product 2h

$^{1}$HNMR (600 MHz, CHLOROFORM-D) δ 8.13 (s, 1H), 7.45 (d, *J* = 7.1 Hz, 2H), 7.38 (t, *J* = 25.5, 7.2 Hz, 3H), 5.38 (s, 2H).

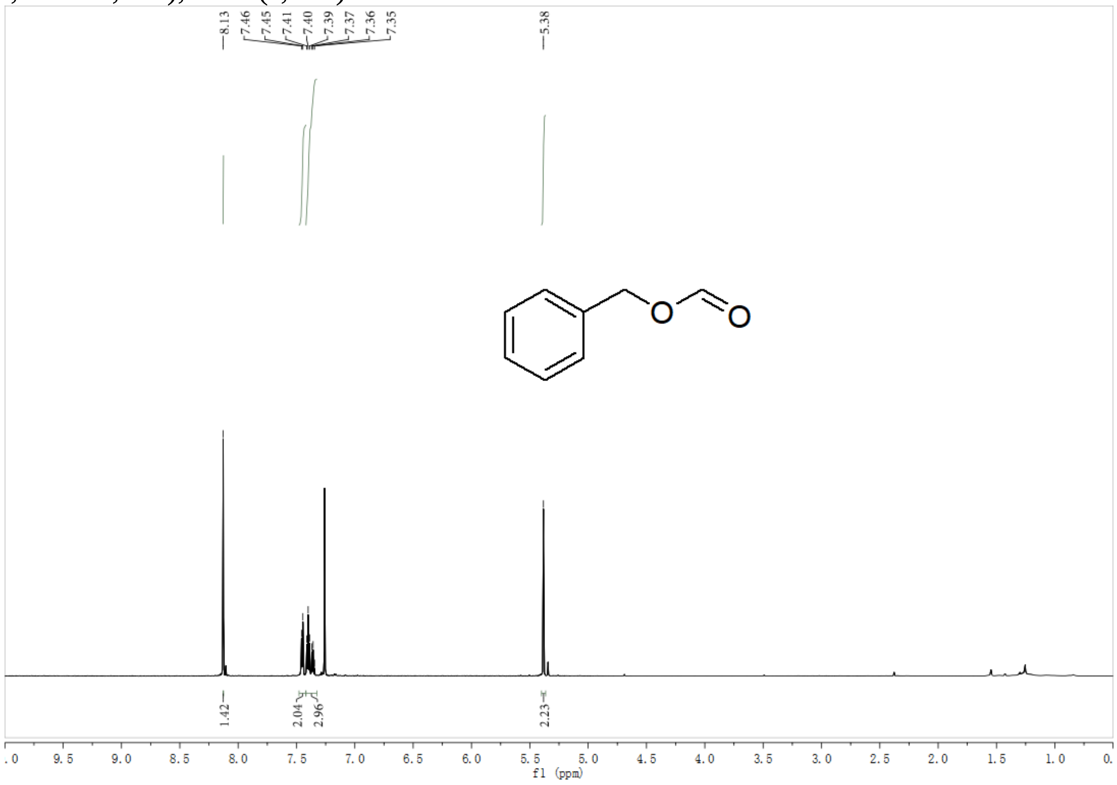

Supplement: Document S1. Transparent Methods, Figures S1–S8, and Table S1 [file mmc1.pdf]
